# Supplementary material for: Can holographic optical storage displace Hard Disk Drives?
Source: Commun Eng. 2024 Jun 18;3:79. doi: 10.1038/s44172-024-00225-0 (PMC11189537; doi:10.1038/s44172-024-00225-0)
Supplement: Supplementary file 2 — Supplementary Information [file 44172_2024_225_MOESM2_ESM.pdf]

Supplementary Information:

Can holographic optical storage displace Hard Disk Drives?

Jiaqi Chu[1], Nathanaël Cheriere[1], Grace Brennan[1], Mengyang Yang[1], Greg O'Shea[1], Jannes Gladrow[1], Douglas J. Kelly[1], Giorgio Maltese[1], Alan Sanders[1], Dushyanth Narayanan[1], Benn Thomsen[1], Antony Rowstron[1]

[1] Microsoft, 198 Science Park, Milton Road, Cambridge CB4 0AB, UK

Correspondence to Jiaqi Chu[1], jiaqchu@microsoft.com

Supplementary Note 1: Methods of diffraction efficiency measurement and confirmation of linearity

The experiments presented in this work fall within the linear low light regime. We validated the linearity across write, write erasure and read erasure processes. The validation gives credibility to our choice of fluence as the horizontal abscissa, which is used for later energy profile optimization.

In the data page experiments, we measured diffraction efficiency using an Ximea CB500MG-CM camera. When acquiring the data points, we performed reads at every 0.001° around the write angle, with a range of ±0.005° (these are the angles at the mirror plane). The maximum diffraction efficiency observed in this range was taken as the result. The scan of the read angle around the write angle was to ensure that we accounted for the impact of lab stability on measurement of diffraction efficiency at the specific angle.

In the write experiment (Supplementary Figure 1a), we delivered a designed amount of energy and write one data page at a fixed angle in a fresh crystal (reset by UV erasure) and measured the maximum diffraction efficiency across 11 scanned angles around the angle where the hologram was written. Then we erased the crystal using UV light from a LUMINUS SBM-120-UV LED. The total write power was controlled via an acoustic optic modulator, with an optimized ratio of 1.7 between the reference and signal beam power. As shown by the overlapping curves in Fig S1a, where we varied the incident power whilst adjusting the exposure time to keep the fluence constant, the crystal Fe0.03:r0.08 demonstrated a linear response to the incident power with a write efficiency A_s/τ_r of approximately 0.04 cm^2/J. In this measurement, we minimized the read energy used for diffraction efficiency reading, and it was ignored during calculation of write efficiency.

In the write erasure experiment (Supplementary Figure 1b), we first wrote a data page as a reference at a specific angle of the reference beam, using a total energy of 14mJ. Subsequently, we wrote a series of data pages at incremental angles, each with 21mJ energy, to simulate the writing of multiple holograms, in the same zone. After writing the later holograms, we read the diffraction efficiency of the first reference data page. Fig S1b shows the how the diffraction efficiency of the reference hologram degrades as a function of the accumulated fluence of the subsequent writes. In this dataset, the write erasure constant τ_e was approximately 210J/cm^2, and the degree of stretch is 0.41. We also conducted this experiment at different incident powers to confirm the linearity as shown by the overlapping traces.

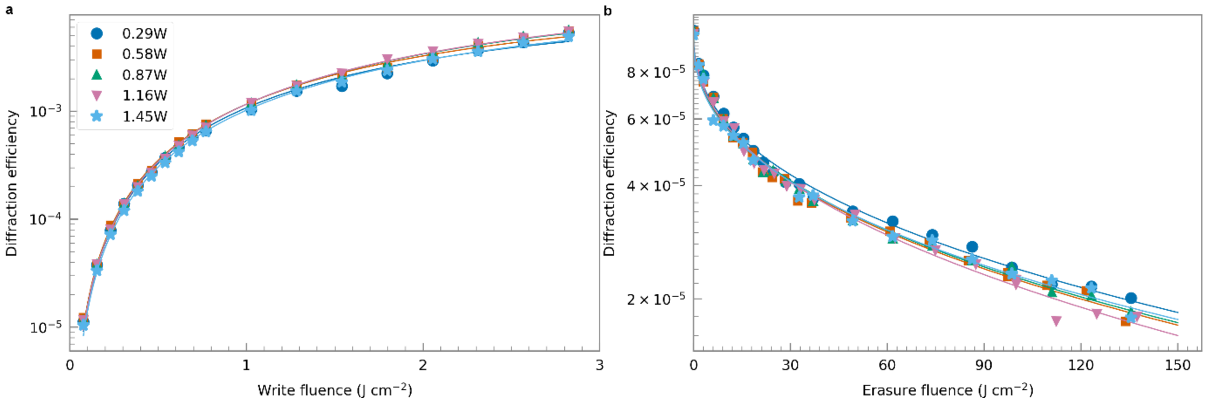

Supplementary Figure 1| Overlapping of the curves at different incident powers indicates a linear relationship between illumination time and write/erasure intensity. a, Diffraction efficiency of single hologram as a function of write fluence used to write the hologram. **b,** Impact of write erasure on test hologram as a function of the Fluence used to write additional holograms in the same volume. In **a** and **b** the various datasets represent experiments conducted with various power.

Supplementary Note 2: Measurement of minimum acceptable energy e_c at camera

The minimum acceptable energy at camera is determined by signal to noise ratio per symbol that enables a BER below a threshold. To measure this, we first used a 47kB signal beam data page and calibrated the average pixel amplitude in relation to beam power under various fixed camera reading settings (Supplementary Figure 2a). In these experiments, the camera exposure was set at 1ms. Subsequently, we decoded the read data pages and worked out the relationship between BER and the energy received by the camera (Supplementary Figure 2b). The result shows that, to achieve a BER of less than 0.01, the minimum energy received by the camera should be higher than 30nJ for use of Ximea CB500MG-CM for a 47KB data page with a gain of 25.

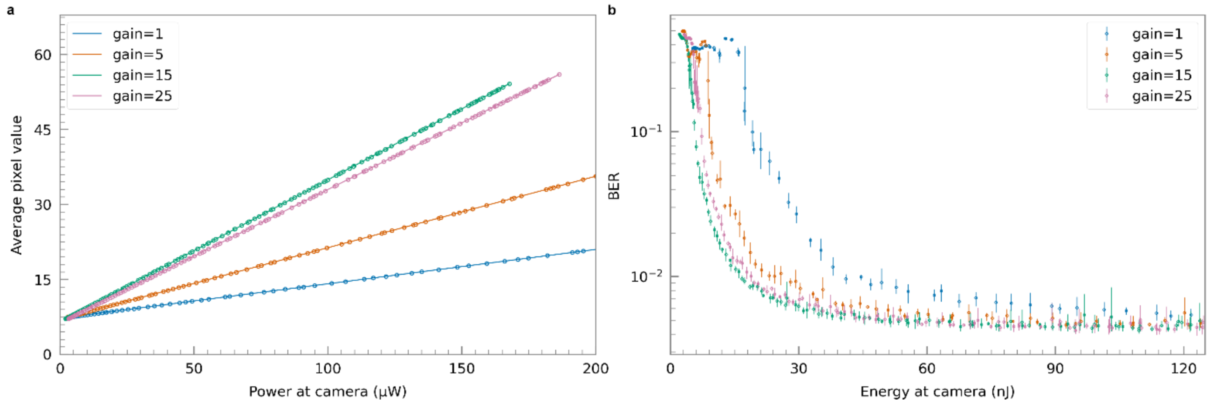

Supplementary Figure 2| Measurement of energy at camera. a, Average pixel value as a function of received power at fixed camera exposure time of 1ms. **b,** BER as a function of energy arriving at camera.

Supplementary Note 3: Computing net IO/J from the profiles

To compute our metric of interest we need to be able to compute the overhead induced by the garbage collection and refresh mechanisms. This is achieved by running an event-driven simulator that simulates the IO on the storage device, which includes running the garbage

collector and the refresh mechanism. While running a workload simulation, we count the number of writes and reads that are directly serving a user's request and the overhead of the system. Net IO/J and raw IO/J are computed from these values and the read and write profiles.

The curves showing the best trade-offs between number of pages and net IO J^{-1} (such as Fig. 4d-e and Fig. 5c-d) are obtained by using an optimization algorithm: NSGA-II, a genetic algorithm. This optimization finds the parameters of the profile generation (the number of pages per zone, and the energy of the last write – as described in section Optimization of energy profiles) and the parameter of the simulator (the number of reads that can be done in a zone before the data needs to be refreshed) that will give interesting trade-offs between the net IO/J and the average amount of data stored per zone.

Supplementary Note 4: Details in demonstrations of number of reads and the density

In the experiments aimed at demonstrating energy efficiency and the number of reads, we angularly multiplexed 400 47KB pages with a volume of 15.288mm^3 in the crystal Fe0.015:r0.04 (dimensions: 2.1 x 2.6 x 2.6mm). We selected this crystal due to its slow erasure properties, making it suitable for studying erasure dynamics. The writes were done over range of angles between reference beam and signal beam from 79.86° to 94.6° with a separation of 0.04° between adjacent pages. The central range of angles 88-92° was not used, as interference caused by back reflections made this region unsuitable for hologram writing. To ensure the hologram was readout at the optimum angle each hologram was read by taking 10 images scanned from -0.01° to +0.01° every 0.002° in air, around the write angle, and the image with the maximum diffraction efficiency was decoded. The dataset with 100% reads (Supplementary Figure 3b) had worse BER than the dataset with 50% reads (Supplementary Figure 3a). Out of the 12672 total reads, 9306 had a BER that was below 0.1 and correctable with the LDPC error coding that was used in this system [1]. This resulted from the uncertainty in the material parameters in more complicated experiments, as well as the build-up of noise and worse lab stability issues over long experiments durations.

In the demonstration of net density, we angularly multiplexed 705 pages in a 5.376mm^3 volume, with a raw page size of 128.25KB, in the crystal Fe0.03:r0.08. The number of pages was constrained by the numerical aperture of the reference beam relay lenses. The usable range of angles between reference beam and signal beam were limited to 84.73-95.54° in crystal with the central region (88-92°) again not used. Adoption of a new decoding pipeline that leverages machine learning (ML) improved the BER and increased the decode rate by over threefold when decoding 2-bit data pages, compared to our conventional pipeline. Lens aberrations constrained us to a maximum BER of 0.09 (solid dots in Supplementary Figure 3c), resulting in a minimum decode rate of 57.2%, and a net density of 9.6GB/cm^3 (assuming every page is decoded with the minimum decode rate). For context, the BER resulting from conventional pipeline is too high to permit effective error correction.

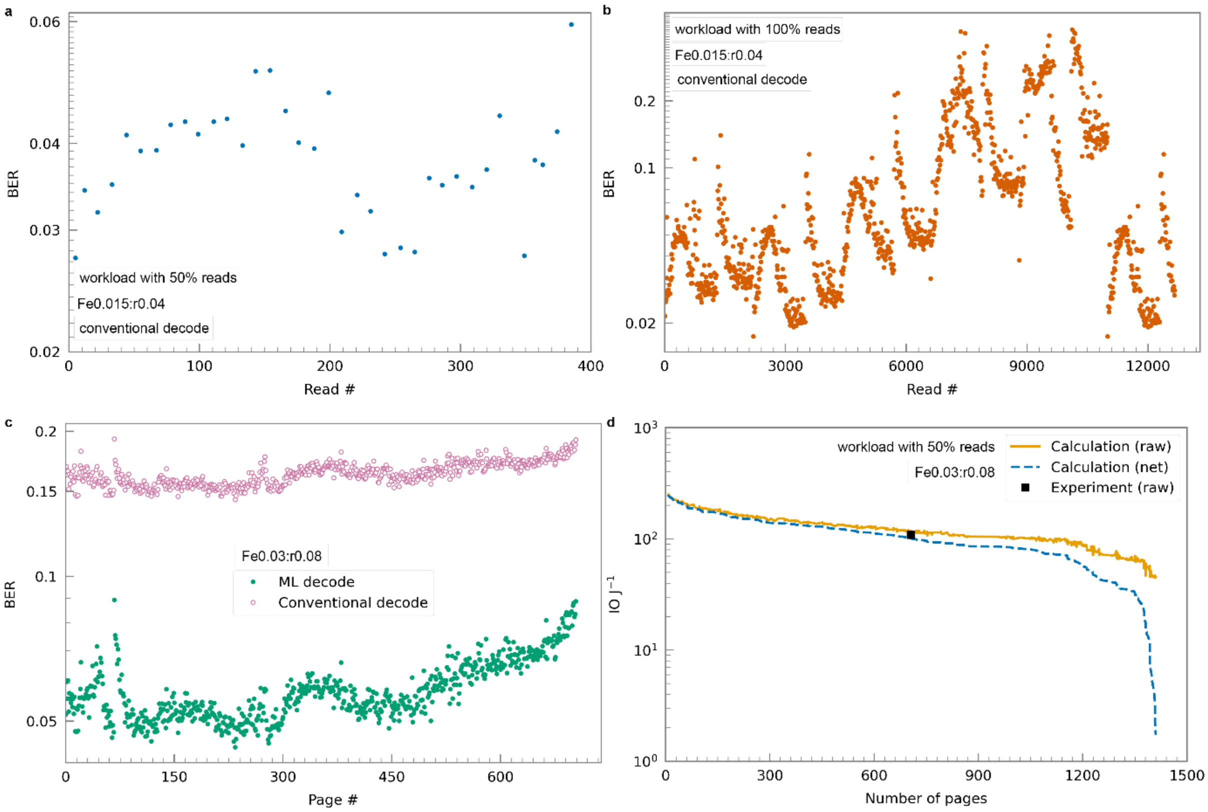

Supplementary Figure 3| BER in the demonstrations. a, BER (log y axis) of a dataset with 400 writes and 399 reads. **b,** BER (log y axis) of a dataset with 400 writes and 12679 reads. **c,** Demonstration of net density: BER (log y axis) of 705 multiplexed pages decoded by machine learning (ML) pipeline and conventional decode, respectively. **d,** Energy efficiency of the density experiment (log y axis). The solid curve represents raw energy efficiency, while the dashed curve illustrates the net energy efficiency which considers garbage collection and refresh. The square marker indicates the raw energy efficiency in the density experiment.

Supplementary Note 5: Estimate best energy efficiency using one-centre charge transport model in combination with profile optimization

A detailed derivation for the application of one-centre charge transport model in the 90° geometry can be found in e.g. [2]. The model itself is explicit, but complexities arise when allocating the physical parameters. We have used the values from [2-6] in this work. The write efficiency is most sensitive to the Glass constant, and we use a value of 3×10^{-9}cm/V estimated from [3]. This value fits our experimental diffraction efficiency for most of the crystals but does not align well with the measurements from the highly reduced crystals. The erasure constant is most sensitive to the photoconductivity, and we learn this value from [5] by fitting the measurements presented in this reference. The one-centre model predicts an exponential decay, which does not account for the observed stretch in our experiments.

We search the best case of energy efficiency versus the number of pages for each doping level by sweeping across a range of Fe^{2+} concentration. We model the write efficiency and erasure constants given the doping level and Fe^{2+} concentration with the one-centre model. These calculated parameters are then integrated into workload-aware optimization of write/read profiles. For each doping level, we find the best Fe^{2+} concentration, using a threshold of 100 net IO J^{-1} to determine the achievable number of pages. Fig. 5e provides an estimate of the best

performance across different doping levels. This data underestimates compared to the experiments partly because the model does not consider the stretched-exponential decay.

Supplementary Note 6: Energy efficiency analysis for co-doped crystals

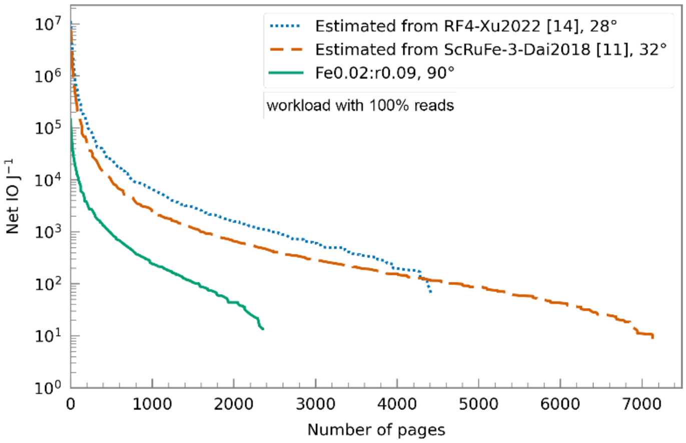

Supplementary Figure 4| Calculated energy efficiency for workloads with 100% reads at e_c=30nJ in co-doped crystals inferred from literature.

Supplementary Note 7: Impact of minimum acceptable energy at camera

Further improvements in energy efficiency and the number of pages could be achieved by reducing the energy at camera. As shown by Supplementary Figure 5a, a 2x improvement in energy efficiency could be achieved in the case of a workload with 50% reads, and a 6x improvement could be achieved in the case of a workload with 100% reads if the energy at the camera e_c was reduced from 30nJ to 5.8nJ. With reduced e_c it could also be possible to increase the number of multiplexable pages to 4500 at 100 net IO/J using the crystal Fe0.02:r0.09, which has been tested, rather than needing to resort to a higher Fe doped crystal with an ideal oxidation state (Supplementary Figure 5b).

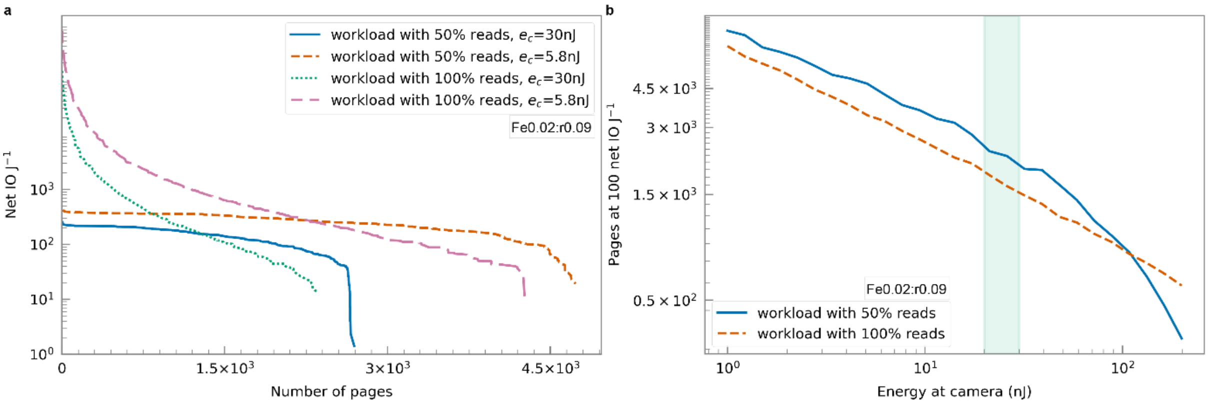

Supplementary Figure 5| Minimum acceptable energy. a, Net IO/J (log scale) at 5.8nJ energy at camera in comparison to that at 30nJ. **b**, The multiplexable number of pages (log scale) threshold at 100 net IO/J as a function of energy at camera (log scale).

Supplementary Note 8: Characterization of reliability

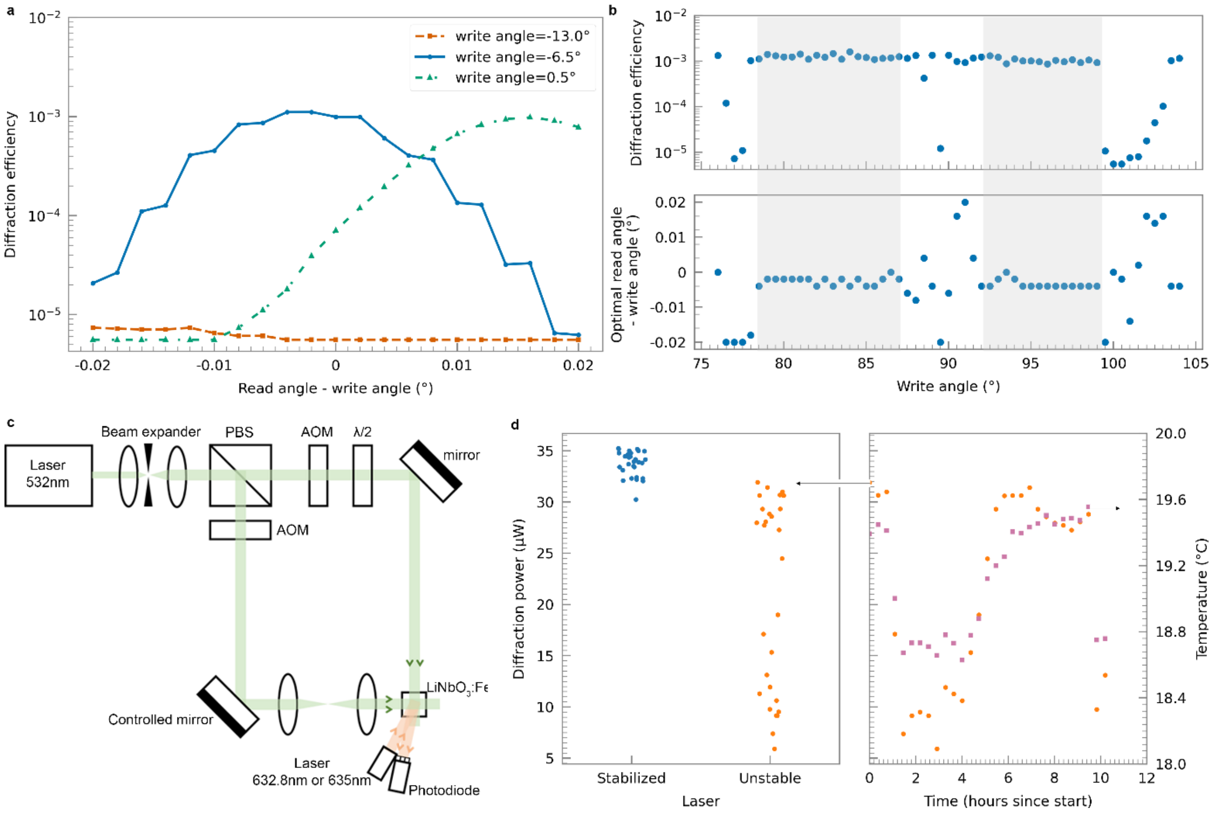

Supplementary Figure 6|Characterization of reliability. a, Example of an ideal write angle aligning with its optimal read angle, contrasted with non-ideal write angles showing shifts in optimal read angle or lacking an optimal read angle. **b**, Identification of ideal write angles for best diffraction efficiency and minimal read angle shift. **c**, Schematic of a media characterization rig that measures the diffraction efficiency of holograms as they are written. PBS stands for polarized beam splitter. AOM stands for acousto-optic modulator. λ/2 stands for half-wave plate. **d**, Repeatability of diffraction power measured by a photodiode. Variations in power from an unstable readout laser corresponded with temperature fluctuation.

Supplementary References

[1] LDPC error coding: *openairinterface5g/openair1/PHY/CODING/nrLDPC_decoder/doc/nrLDPC/nrLDPC.pdf at master · oai-group/openairinterface5g · GitHub*

[2] Burr, G. W. *Volume holographic storage using the 90° geometry.* Dissertation (Ph.D.), California Institute of Technology. Doi:10.7907/0vbj-pb92 (1996).

[3] Volk, T. & Wöhlecke, M. *Lithium Niobate: Defects, Photorefraction and Ferroelectric Switching* (Springer, 2008).

[4] Shah, R. R., Kim, D. M., Rabson, T. A. & Tittel, F. K. Characterization of iron-doped lithium niobate for holographic storage applications. *J. Appl. Phys.* **47**, 5421-5431 (1976).

[5] Peithmann, K., Wiebrock, A. & Buse, K. Photorefractive properties of highly-doped lithium niobate crystals in the visible and near-infrared. *Appl. Phys. B* **68,** 777-784 (1999).

[6] Ciampolillo, M. V. et al. Quantification of Iron (Fe) in Lithium Niobate by optical absorption. *Appl. Spectrosc.* **65**, 216-220 (2011).
