# Supplementary material for: Can holographic optical storage displace Hard Disk Drives?
Source: Commun Eng. 2024 Jun 18;3:79. doi: 10.1038/s44172-024-00225-0 (PMC11189537; doi:10.1038/s44172-024-00225-0)
Supplement: Supplementary file 3 — Description of Additional Supplementary Files [file 44172_2024_225_MOESM3_ESM.pdf]

Description of Additional Supplementary Files

File name: Supplementary Data 1

Description: Source data to plot all figures in the main text and Supplementary Information
